# What's different about *Nursing Open?*

**DOI:** 10.1002/nop2.17

**Published:** 2015-04-06

**Authors:** Roger Watson

The first issue of *Nursing Open* was successfully published in 2014, and early in 2015, we are pleased to introduce this second issue. The copy flow is good and *Nursing Open* is clearly being recognized as a reputable place to publish. Of course, we have the support of an excellent publishing house, a distinguished team of associate editors and a prestigious international editorial board. We are also growing our team of reviewers and hope to maintain a good turnaround time for manuscripts.

I emphasize reputation and the nature of our team as these are some of the hallmarks of any good journal; in these days of the exponential growth of online open access journals, reputation is crucial. As I mentioned in my first editorial (Watson 2014), we all receive a large volume of emails inviting us to participate in various capacities with a range of online journals. Not all of these journals are legitimate or honest ventures in the view of Jeffrey Beall in his blog Scholarly Open Access. There has also been considerable interest in the issue of how trustworthy online open access journals are in *Journal of Advanced Nursing* in two editorials titled, respectively, ‘Open access a new frontier’ (Watson *et al*. 2012) and ‘Authors and readers beware of the dark side of open access’ (Pickler *et al*. 2014). This is in addition to attention to the issue by the Committee on Publication Ethics (COPE), in the BMJ blog where Clark (2015) published: ‘How to avoid predatory journals – a five point plan’ and in *Nurse Author & Editor* where Owens (2015) published ‘More trends in predatory publishing practices’.

If you have any worries about online open access publishing then all of the above are worth reading. Also, there is a Directory of Open Access Journals which outlines the basic standards to be met before being listed. *Nursing Open* meets these minimal criteria and is applying to be listed.

Globally, the pressure to publish online open access is increasing. For example, in the UK, the Higher Education Funding Council for England (2014) and its counterparts in the other three countries of the UK have specified that from April 2016 all research outputs to be considered for research assessment have to be published online open access. Simultaneously, Research Councils UK (2014) issued a policy whereby research which they fund has to be published online open access. The Australian Research Council has had an open access policy for research it funds since 2013. Therefore, we expect to see this trend reflected in the pages of *Nursing Open*. For those who would like to know more about Open Access policies, including funder mandates, article publication charges, waivers and discounts, and institutions with funder accounts, information and resources are available at Wiley Open Access.

I hope you enjoy this second issue of *Nursing Open* and that you find the latest articles interesting and useful. I highlight all of the articles in the *Nursing Open* blog where you can read the latest entries on Why do Chinese nurses leave practice? (Zhu *et al*. 2014) and Machine translation and ethnic diversity (Taylor *et al*. 2015); you can also follow us on Twitter.
